# The Preparation, Properties, and Characterization of Octenyl Succinic Anhydride-Modified Turmeric Starches and Their Emulsification for Pickering Emulsions

**DOI:** 10.3390/foods14071171

**Published:** 2025-03-27

**Authors:** Lijuan Fu, Hongfei Chi, Hang Wei, Biao Huang, Yueyue Qiang, Mengzhu Shi, Ling Fang, Jianwei Fu

**Affiliations:** 1 Fujian Key Laboratory of Agro-Products Quality and Safety, Institute of Quality Standards & Testing Technology for Agro-Products, Fujian Academy of Agricultural Sciences, Fuzhou 350003, China; fulijuan_f@163.com (L.F.); chf0825@163.com (H.C.); 13696862957@163.com (H.W.); banbanhb1981@163.com (B.H.); qyydsw@163.com (Y.Q.); mengzhu611@163.com (M.S.); flonly1188@foxmail.com (L.F.); 2 College of Horticulture, Fujian Agriculture and Forestry University, Fuzhou 350002, China; 3 College of Food Science, Fujian Agriculture and Forestry University, Fuzhou 350002, China

**Keywords:** turmeric starches, OSA-modified turmeric starches, degree of substitution, Pickering emulsions

## Abstract

Turmeric has extensive applications in various fields, including food and medicine. However, research on turmeric starch remains relatively scarce. There is a significant lack of in-depth studies on its processing properties and starch modification abilities. In this context, octenyl succinic anhydride (OSA)-modified turmeric starches (O-MTSs) were synthesized. Subsequently, a comprehensive investigation was carried out, including property analysis, characterization, and evaluation of the emulsifying capacity. The alterations in solubility, swelling degree, syneresis, and transparency of turmeric starches before and after modification were systematically studied. The characterization of O-MTSs was conducted using a scanning electron microscope (SEM), particle size analysis, X-ray diffraction (XRD), Fourier transform infrared spectroscopy (FT-IR), and thermogravimetric analysis. The possibility of using O-MTS as an emulsifier to prepare Pickering emulsions was explored. The results show that O-MTS had better solubility, swelling degree, syneresis, and transparency compared to turmeric starches (TSs). The O-MTS retained a relatively intact morphology, but its particle size slightly increased, and the characteristic peak at 995 cm^−1^ shifted to some extent. The relative crystallinity decreased from 32.59% to 18.39%, and the water-binding capacity of O-MTSs improved accordingly. O-MTSs could better stabilize Pickering emulsions as an emulsifier compared to TSs. With the increase in the degree of substitution (DS) and concentration of the O-MTS, its emulsification index (EI) demonstrated an upward trend.

## 1. Introduction

Turmeric, a dried rhizome of the *Curcuma longa* L. plant in the ginger family, has both medicinal and edible values and is widely cultivated in tropical and subtropical regions. In the modern food industry, it serves not only as a versatile seasoning but also as a widely used food pigment, thus possessing high economic value. In traditional Chinese medicine, turmeric is believed to promote blood circulation and clear the meridians. In recent years, research has revealed that turmeric exhibits anti-cancer [1], anti-inflammatory, antibacterial [2], and antioxidant [3] properties, as well as anti-myocardial injury [4], showing broad prospects for clinical applications.

In contemporary research, the focus on turmeric primarily centers around small-molecule active substances, including curcumin (Cur) and turmeric essential oil (TEO). Previous studies have found that encapsulating Cur with soy protein isolate (SPI) and pectin (PE) can improve its photostability and thermal stability. SPI-Cur-PE exhibits a lower release rate and significant scavenging activities of 2,2′-Azino-bis (3-ethylbenzothiazoline-6-sulfonic acid) (ABTS) and 1,1-Diphenyl-2-picrylhydrazyl (DPPH) radicals [5]. Meanwhile, the exploration of using β-cyclodextrin (β-CD) inclusion technology to enhance the stability and controlled release of TEO has been carried out, and the release behavior and kinetics of TEO in β-CD during long-term storage under different relative humidity and temperature conditions have also been investigated [6]. However, these studies mainly revolve around the small-molecule active components in turmeric, with little attention paid to the development and utilization of starch, another major component of turmeric.

Turmeric starch (TS) accounts for approximately 10–20% of the fresh turmeric, yet its current development and utilization are not extensive. Relevant research is mostly confined to the study of some physicochemical properties, with extremely limited research on the modification of turmeric starch. Compared with other common starches like corn starch and pea starch, TS has significant differences in terms of uses, physicochemical properties, functional activities, and nutritional value. TS not only has the basic functions of other common starches, such as thickening, gelatinization, film-forming, and slurry-making, but also contains a relatively high content of resistant starch that is not easily digested and absorbed by the human body [7]. This helps with weight control, improves intestinal health, and prevents chronic diseases. Moreover, due to the presence of curcumin, TS has unique bioactive components with antioxidant and anti-inflammatory properties, which are absent in other common starches [5]. Therefore, the research on the resource utilization of TS is of great significance.

The novelty of this study lies in the fact that it is the first to focus on the turmeric starch resources in the remaining residues after extracting curcumin and turmeric essential oil, as well as its in-depth research on them. Almost no previous research has explored the development and utilization of turmeric starch from this perspective. We innovatively extract turmeric starch from the residues left after extracting small-molecule active substances, thereby achieving the full utilization of turmeric resources and opening up a new direction for turmeric research.

Octenyl succinic anhydride (OSA) is an anhydride that is used to modify starches. It was successfully developed and patented by Caldwell and Wurzburg in 1953 [8]. In an alkaline environment, starch reacts with OSA through an esterification reaction to form octenyl succinic acid starch ester. Through this esterification reaction, hydrophilic carboxyl groups and hydrophobic long-chain alkyl groups are introduced into the starch molecules, endowing the starch with amphiphilicity [9]. As a result, OSA-modified starches have many unique properties compared to natural starches, broadening their applications in industrial production and other fields, such as being widely used as emulsifiers and microcapsule wall materials in the food, cosmetics, and pharmaceutical industries.

Pickering emulsion is a type of emulsion stabilized by micron- or nanosized solid particles instead of traditional surfactants [10]. Common solid particles include inorganic particles such as silica and calcium carbonate, as well as organic particles like starch and cellulose. These particles adsorb at the oil–water interface, forming a physical barrier and interacting with the interface through van der Waals forces, electrostatic forces, etc., which provides strong stability [11]. In comparison, classical emulsions rely on the amphiphilicity of surfactant molecules to reduce the interfacial tension between oil and water, forming an oriented molecular film that stabilizes the emulsion [12]. The droplets of Pickering emulsions are large, are in the micron range, and have a wide distribution because the solid particles affect the packing at the interface and the wrapping effect [13]. The droplets of classical emulsions are small, are in the nanometer-to-submicron range, and have a uniform distribution, as the surfactants can reduce the interfacial tension uniformly. In terms of stability, Pickering emulsions demonstrate good long-term stability and strong tolerance to high temperatures, high salt concentrations, etc. [13]. When the temperature, pH value, or electrolyte concentration changes, the performance of surfactants in classical emulsions will be affected, and they are prone to demulsification and stratification. In this study, esterified and modified turmeric starch is selected as the solid particle for preparing the Pickering emulsion. The influence of esterified and modified turmeric starch on the emulsification index of the emulsion during the preparation process of Pickering emulsions is explored in depth to evaluate its application potential in the field of Pickering emulsion preparation.

The objective of this study is to extract turmeric starch from the residues obtained after curcumin and turmeric essential oil extraction and then carry out OSA esterification modification to prepare O-MTS. The preparation process parameters will be optimized through single-factor experiments and orthogonal tests, and O-MTS will be characterized by SEM, XRD, and FT-IR. By measuring the physicochemical and thermal properties of O-MTS, the effects of different degrees of substitution (DS) on the properties of O-MTS will be explored. Using O-MTS as an emulsifier to prepare Pickering emulsions, the factors affecting the emulsifying properties of Pickering emulsions will be analyzed.

The significance of this study is two-fold. On one hand, it determines the in-depth development and comprehensive utilization of turmeric resources, thereby increasing the added value of turmeric. On the other hand, through the modification research of turmeric starch, new approaches and theoretical bases for its applications in the food, medicine, and cosmetics fields are provided and are expected to promote the development of turmeric-starch-related industries. Meanwhile, this study also provides references for the modification and comprehensive utilization of other plant starches.

## 2. Materials and Methods

### 2.1. Chemicals and Materials

OSA was purchased from Aladdin Reagent Co., Ltd. (Shanghai, China). Sodium hydroxide, silver nitrate, and phenolphthalein were purchased from McLean Biochemical Technology Co., Ltd. (Shanghai, China). Isopropyl alcohol and absolute ethanol were purchased from the National Pharmaceutical Group Chemical Reagent Co., Ltd. (Shanghai, China). Soybean oil was purchased from Yihai Kerry Arawana Holdings Co., Ltd. (Shanghai, China).

### 2.2. Preparation and Process Optimization of Octenyl Succinic Anhydride-Modified Turmeric Starches (O-MTSs)

The O-MTSs were prepared according to the method of Zhang et al. [14]. TS of a certain concentration was prepared and stirred with a magnetic stirrer (WH-260R, WIGGENS, Beijing, China) to control the reaction temperature and stirring speed required for modification. After stirring for 10 min, the system was adjusted to a certain pH value with 3% NaOH, and then OSA diluted with anhydrous ethanol 5 times was added. During this process, the pH value and reaction temperature of the reaction system were kept stable at the preset levels. After the reaction was complete, 2 mol/L of HCl was used to adjust the pH of the reaction system to close to 6.5 to end the reaction. The O-MTS was washed three times with distilled water, followed by ethanol, and then distilled water again. Subsequently, it was dried in an oven at 45 °C for 24 h. After that, it was ground to obtain the final O-MTS product.

The effects of different OSA additions (1%, 3%, 5%, 7%, and 9%), reaction times (2 h, 3 h, 4 h, 5 h, and 6 h), reaction temperatures (30 °C, 35 °C, 40 °C, 45 °C, and 50 °C), starch paste concentrations (25%, 30%, 35%, 40%, and 45%), and pH (7.5, 8.0, 8.5, 9.0, and 9.5) were investigated on the esterification reaction by using the DS of O-MTSs as an indicator. Based on the results of the single-factor experiment, we selected the points with higher DS values for further experimentation and designed an L_9_(3^4^) orthogonal test table to study the effects of TS concentration, reaction time, reaction temperature, and pH on the esterification reaction. Thus, the optimal preparation process parameters for O-MTS were obtained (see the detailed experimental methods and results in the Supplementary Materials).

### 2.3. Determination of Degrees of Substitution (DS)

The DS of O-MTS was determined using the titration method [14]. An amount of 5 g O-MTS was added to the 5 mL HCl–isopropanol solution (2.5 mol/L), and the mixture was stirred with a magnetic stirrer for 30 min. Then, 100 mL of the 90% isopropanol solution was added, and the mixture was stirred for an additional 10 min. The solution was filtered by suction and washed with 90% isopropanol until no Cl- was detected (the solution remained clear) upon testing with the 0.1 mol/L silver nitrate solution. After that, it was diluted to 300 mL with distilled water. Then, it was heated in a water bath for 30 min with intermittent shaking. Phenolphthalein was used as the indicator, and titration was performed with a 0.1 mol/L NaOH solution. The TS was used as blank control. The calculation formula for DS is given as follows:

(1) $$DS=\frac{0.162\times (A\times M)/W}{1-[0.210\times (A\times M)/W]}$$

In the formula, the following variables are used:

A—the volume of consumption of 0.1 mol/L NaOH standard solution for titration, mL;

W—dry mass of O-MTS, g;

M—concentration of NaOH standard solution, mol/L.

### 2.4. Determination of Solubility and Swelling Degree

Here, we referred to the method of Gong et al. [15], with slight modifications. A 2.0% TS liquid was prepared and placed in water bath pots (DHG-9070A, Shanghai Hongdu electronic technology Co., Ltd., Shanghai, China) at 55 °C, 65 °C, 75 °C, 85 °C, and 95 °C, heated in a water bath for 30 min, and taken out and shaken every 5 min. After that, it was cooled to room temperature, and then a centrifuge (TDL-5-A, Shanghai Anting scientific instrument factory, Shanghai, China) was used to spin it at 3000 r/min for 15 min. The supernatant was poured into a glass Petri dish with a known mass in advance, dried in a blast drying oven to a constant weight, and weighed and recorded as C (g). The mass of the precipitate in the centrifuge tube was B (g). Finally, the solubility and swelling degree of TS was calculated. The calculation method is shown as follows.

(2) $$S(\%)=\frac{C}{A}\times 100$$

(3) $$D(\%)=\frac{B}{A(100-S)}\times 100$$

In the formula, the following variables are used:

S—solubility, %;

C—the amount of substance precipitated from the supernatant, g;

A—mass of starch sample taken, g;

D—degree of swelling, %;

B—mass of precipitate in the centrifuge tube, %.

### 2.5. Determination of Thaw Restability

The method was adapted from that of Zhou et al. [16], with minor modifications. TS and O-MTS were taken and prepared into a liquid with a concentration of 6.0%, which was then heated and stirred in a 90 °C water bath for 30 min. A mass of the starch paste, designated as M1 (g), was placed into a centrifuge tube, and after being stored in a −18 °C freezer for 24 h, it was removed for natural thawing. The tube was then centrifuged at 3000 r/min for 15 min, the supernatant was removed, and then the precipitate mass M2 (g) was weighed. The syneresis rate was finally calculated as follows:

(4) $$P(\%)=\frac{M1-M2}{M1}\times 100$$

In the formula, the following variables are used:

M1—mass of the starch paste, g;

M2—mass of the precipitate in the centrifuge tube, g.

### 2.6. Determination of Transparency

Here, we referred to the method of Shang et al. [17], with slight modifications. A 1% TS liquid was prepared, heated, and stirred in a 90 °C water bath for 30 min. Then, it was cooled to room temperature, and finally, a spectrophotometer (TP-214, DENVER, Denver, CO, USA) was used to measure the absorbance of the sample liquid at 600 nm. Distilled water was used as the blank group.

### 2.7. Characterization of Octenyl Succinic Anhydride-Modified Turmeric Starches (O-MTSs)

#### 2.7.1. SEM Analysis

Following the methodology proposed by Zhang et al. [18], the O-MTS powder was evenly dispersed on the SEM sample stage with a conductive adhesive. Subsequently, a gold plating treatment was carried out. The surface morphology of the O-MTS was then observed at a magnification of 2000× using a scanning electron microscope (JSM-6380LV, JEOL Ltd., Showima City, Japan).

#### 2.7.2. Particle Size Analysis

According to the method of Sun et al. [19], the particle size of samples was analyzed using a particle size analyzer (NanoPlus3, Micromeritics, Norcross, GA, USA). Samples (0.1 g) were suspended in 10 mL of double-deionized water and mixed well. A semiconductor laser with a wavelength of 680 nm was used as the light source with an output power of 3 mW, and the laser diffraction and scattering intensity patterns were measured within a range of 0.017–2500 μm.

#### 2.7.3. Fourier Transform Infrared Spectroscopy (FT-IR) Analysis

According to the method of Fu et al. [5], the potassium bromide powder was mixed with the samples in a ratio of 100:1, and then the mixture was pressed into a sheet using a presser. First, a pure potassium bromide sheet was put into the instrument for scanning as a reference, and then the prepared sample sheet was scanned. The parameters were a spectral resolution of 4 cm^−1^, a measurement range from 400 cm^−1^ to 4000 cm^−1^, and 16 signal scan accumulations.

#### 2.7.4. X-Ray Diffractometer (XRD) Analysis

According to the method of Mao et al. [20], an XRD (DY1602, Malvern Panalytical, Singapore) was used to detect the crystal structure of starch samples. The parameters were set as follows: current, 40 mA; working voltage, 40 kV; scanning speed, 8.000° per min; scanning range, between 5.00° and 40.00°; and scanning step size, 0.02°.

#### 2.7.5. Thermal Gravity Analysis

The procedure used is as follows: Place the sample in the crucible, heat it from 30 °C to 600 °C at a rate of 10 °C/min, and conduct thermogravimetric analysis under nitrogen with a flow rate of 50 mL/min using a thermogravimetric analyzer (Labsolar-6A, German NETZSCH, Selb, Germany).

### 2.8. Preparation of Pickering Emulsions

According to the method of Yu et al. [21], O-MTS was dispersed in 10 mL of deionized water. A high-speed dispersion homogenizer (FJ200-SH, Shanghai Huxi Industrial Co., Ltd., Shanghai, China) was adjusted to 15,000 r/min, and the suspension was homogenized for 30 s. Then, edible oil (in a 1:1 oil–water ratio) was added, and the mixture was homogenized at 20,000 r/min for 20 s. After 1 min, it was homogenized again for 20 s to obtain a Pickering emulsion.

### 2.9. Pickering Emulsion Stability Analysis

#### 2.9.1. The Effect of DS Value on the Stability of Pickering Emulsions

Under the conditions of a 1% concentration of O-MTS particles and a 1:1 oil–water ratio, the Pickering emulsion was prepared. When the DS values of O-MTSs were 0, 0.0144, 0.0188, 0.0229, and 0.0264 (corresponding to OSA addition amounts of 0, 0.5%, 1%, 2%, and 3%), respectively, the effect of DS value on the emulsifying property of the Pickering emulsion was explored.

#### 2.9.2. The Effect of Starch Concentration on the Stability of Pickering Emulsions

Under the conditions of a 1:1 oil–water ratio and a DS value of 0.0264, the Pickering emulsion was prepared. When the particle concentration of O-MTS was 1%, 1.5%, 2%, 2.5%, and 3%, the influence of particle concentration on the emulsification of Pickering emulsion was discussed.

#### 2.9.3. Determination of the Emulsification Index (EI)

We referred to the method of Xiao et al. [22], with appropriate modifications made. The emulsion was transferred to a 10 mL transparent glass bottle, and the sample was observed after being placed at room temperature at different times. The EI was calculated according to the following formula:

(5) $$EI(\%)=\frac{He}{H}\times 100$$

In the formula, the following variables are used:

He—the height of the emulsified layer of the Pickering emulsion, cm;

H—the total height of the Pickering emulsion, cm.

#### 2.9.4. Super-Depth-of-Field Microscope (SDM) Detection

After the Pickering emulsion was left standing for 1 d, 20 μL samples were taken on a glass slide, and an SDM (LEICA, DVM6-A, Wetzlar, Germany) was used to observe the structure of the Pickering emulsion and take pictures (magnification of 400×).

### 2.10. Data Statistics and Analysis

All data were plotted using Origin 2019 software, and one-way analysis of variance (*p* < 0.05) was performed on the experimental data using SPSS 19.0 to determine significant differences. All experiments were repeated three times, and the results were expressed as mean ± standard deviation.

## 3. Results and Discussion

### 3.1. Results of Process Optimization

The results of the single-factor experiment and orthogonal experiment are shown in the Supplementary Materials. The optimal process conditions for preparing O-MTSs are as follows: reaction time, 5 h; reaction temperature, 40 °C; TS concentration, 35%; pH value, 8; and OSA addition amount, 3%. Under these conditions, the degree of substitution reached 0.0282.

### 3.2. Changes in Solubility and Swelling Degree

**Figure 1:**
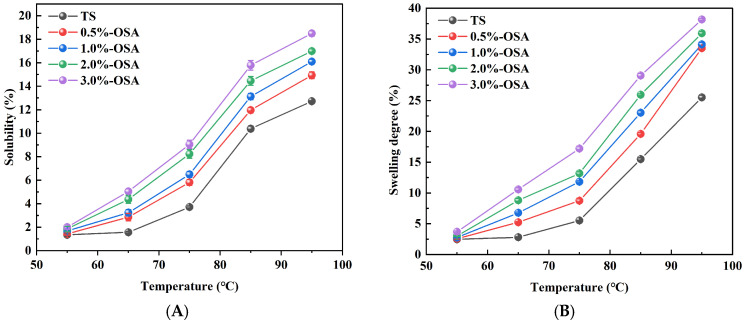
The influence of different amounts of octenyl succinic anhydride (OSA) on the changes in solubility (**A**) and swelling degree (**B**) of octenyl succinic anhydride-modified turmeric starch (O-MTS) is presented. Here, TS is turmeric starch. OSA-0.5% denotes the modified turmeric starch with an OSA addition amount of 0.5%; OSA-1% denotes the modified turmeric starch with an OSA addition amount of 1%; OSA-2% denotes the modified turmeric starch with an OSA addition amount of 2%; and OSA-3% denotes the modified turmeric starch with an OSA addition amount of 3%.

The solubility and swelling degree of starch reflected the extent of interaction between starch and water. The solubility and swelling degree of starch under different OSA amounts and temperatures are shown in Figure 1A,B. The solubility and swelling degree of O-MTS were significantly higher than those of TS (*p* < 0.05), indicating a significant improvement in the dissolution performance of O-MTSs. Due to OSA esterification, the molecular structure of starch loosened. This allowed water molecules to penetrate the starch more easily, diffuse, and spread within the paste [23]. The solubility and swelling degree of O-MTSs increased with the addition of OSA. The carboxylic acid groups introduced onto the starch molecules have strong hydrophilicity, and the interaction between the modified starch and water becomes more intense [9]. With the increase in substitution degree, this interaction force also increases, resulting in greater solubility.

### 3.3. Changes in Freeze–Thaw Stability

**Figure 2:**
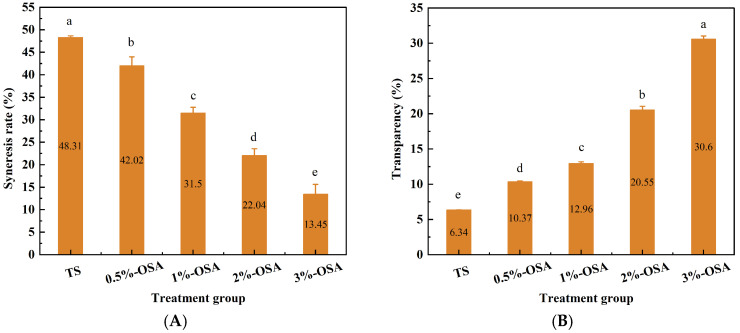
The influence of different amounts of octenyl succinic anhydride (OSA) on the changes in syneresis (**A**) and transparency (**B**) of octenyl succinic anhydride-modified turmeric starch (O-MTS) is presented. Different letters (a–e) indicate significant differences (*p* < 0.05, *n* = 3). Here, TS is turmeric starch. OSA-0.5% denotes the modified turmeric starch with an octenyl succinic anhydride (OSA) addition amount of 0.5%, OSA-1% denotes the modified turmeric starch with an OSA addition amount of 1%, OSA-2% denotes the modified turmeric starch with an OSA addition amount of 2%, and OSA-3% denotes the modified turmeric starch with an OSA addition amount of 3%.

Freeze–thaw stability refers to the stability of starch paste when it undergoes alternating cycles of freezing and thawing. The evaluation index for freeze–thaw stability is the syneresis rate, with a lower syneresis rate indicating better freeze–thaw stability of the starch paste. The syneresis rate after freeze–thaw of O-MTS paste with different OSA additions was shown in Figure 2A. The results showed that the freeze–thaw stability of O-MTS was significantly improved compared with TS (*p* < 0.05). The syneresis rate of TS paste was 48.31%, indicating that its freeze–thaw stability was poor. O-MTS paste exhibited a trend of decreasing the syneresis rate with the increase in OSA addition. When the OSA addition was 3%, the syneresis rate was 13.45%, indicating that the freeze–thaw stability of the O-MTS had been greatly improved. This may be because during the esterification process, carboxyl groups are introduced into the starch molecules, thereby enhancing the interaction between starch and water molecules, making it difficult for water molecules to separate and improving the freeze–thaw stability of starch [9]. The improvement of freeze–thaw stability can also reflect the reduction in retrogradation. After modification, the introduction of OSA increases the spatial effect between starch molecules, preventing the rearrangement of starch molecular chains during the low-temperature storage of O-MTS paste [24]. This characteristic helps O-MTS to be used in frozen foods.

### 3.4. Changes in Transparency

Transparency is one of the most important external characteristics exhibited by starch, directly related to the appearance and application of starch products, which in turn affects the acceptability of these products. As shown in Figure 2B, the transparency of O-MTS was higher than that of TS, reaching 30.6% when the OSA addition was 3%. This indicated that O-MTS helped improve transparency, and the transparency increased with the addition of OSA. This was consistent with Liang’s research findings [25], and it may be because the introduction of carboxyl groups into the starch molecules increased the interaction between starch and water molecules, weakened the association between starch molecular chains, and reduced the degree of light reflection and refraction, thereby improving the transparency of starch solution.

### 3.5. Changes in Particle Morphology

**Figure 3:**
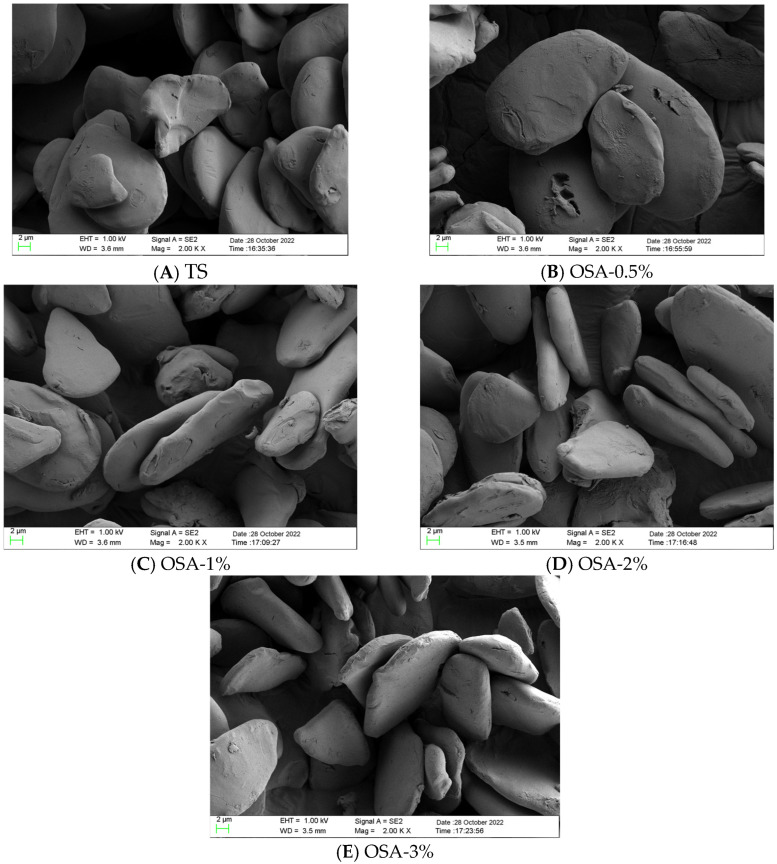
Scanning electron microscopy (SEM) images of turmeric starch (TS) and octenyl succinic anhydride-modified turmeric starch (O-MTS) are presented. In Figure (**A**), the morphology of turmeric starch (TS) is shown. Figure (**B**) depicts the modified turmeric starch with an octenyl succinic anhydride (OSA) addition of 0.5% (OSA-0.5%). Figure (**C**) illustrates the modified turmeric starch with an OSA addition of 1% (OSA-1%). Figure (**D**) exhibits the modified turmeric starch with an OSA addition of 2% (OSA-2%), and Figure (**E**) demonstrates the modified turmeric starch with an OSA addition of 3% (OSA-3%).

As shown in Figure 3, the morphological characteristics of TS and O-MTS were evaluated by SEM with magnifications of 1000× and 2000×. The TS exhibited triangular and elliptic shapes stacked in a pile and had a smooth surface. After OSA modification, the shape of the O-MTS particles remained basically unchanged, still triangular and elliptic, but their surfaces became rough and uneven, and there were many small pores. This indicated that the modification reaction mainly occurred on the surface of the starch particles without affecting their internal structure. The changes in surface characteristics were due to the attack of OSA on the particle surface, resulting in the formation of small pores [26]. Another possible reason is that when NaOH was added to the esterification reaction to maintain alkaline conditions, surface gelation resulted in rough particle surfaces [27]. However, there were no obvious cracks on the surface of O-MTS. Sharma et al. [26] also reported similar results for pearl millet starch, and Abiddin et al. [28] reported similar results for tapioca starch.

### 3.6. Changes in Particle Size Analysis

**Figure 4:**
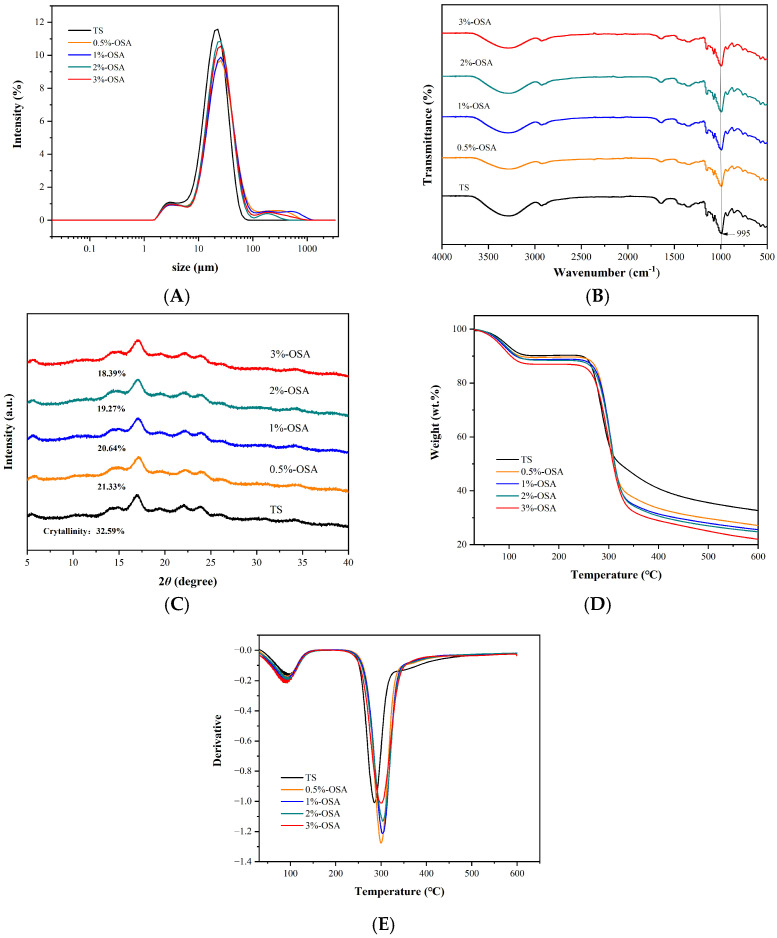
The diagrams of various characterization techniques for turmeric starch (TS) and octenyl succinic anhydride-modified turmeric starch (O-MTS) are presented as follows: (**A**) particle size distribution, (**B**) Fourier transform infrared spectroscopy (FT-IR), (**C**) X-ray diffraction (XRD), (**D**) Thermogravimetric analysis (TGA), and (**E**) derivative thermogravimetry (DTG). Here, TS is turmeric starch. OSA-0.5% denotes the modified turmeric starch with an octenyl succinic anhydride (OSA) addition of 0.5%, OSA-1% denotes the modified turmeric starch with an OSA addition of 1%, OSA-2% denotes the modified turmeric starch with an OSA addition of 2%, and OSA-3% denotes the modified turmeric starch with an OSA addition of 3%.

Changes in starch particle size could greatly affect its gelatinization characteristics, light transmittance, and other physical and chemical properties and are key factors in determining starch quality. As shown in Figure 4A, under the effect of OSA esterification, the particle size of O-MTS increased mainly due to the expansion of aggregation between starch molecules. Simsek et al. [29] studied the particle size distribution of four types of starch, including OSA-esterified rice starch. The particle size of starch increased after OSA esterification. This is because the addition of hydrophobic groups makes starch esters prone to polymerization in aqueous solutions during the esterification reaction.

### 3.7. FT-IR Analysis

FT-IR can measure the position, intensity, and shape of the characteristic peak in starch, thereby obtaining information on the groups and chemical bonds in starch and conduct a qualitative analysis of its structure. As shown in Figure 4B, the FT-IR spectra of TS and O-MTS are similar, confirming the slight structural changes in starch in the modification process. Possible reasons were that the starch contains tightly structured crystalline regions and esterification reactions mainly occur on the surface of the granules, making it difficult to penetrate the interior. In addition, the amount of OSA added was small, and its impact on the polar chemical bonds in O-MTS was not significant. It could be observed that the characteristic peak at 995 cm^−1^ underwent a blue shift, which is typically associated with the stretching vibration of C-O-C bonds in starch molecules [30]. During the modification process of starch, the intensity and position of this characteristic peak may also change, reflecting alterations in the molecular structure of starch. Alternatively, when the moisture content in the starch sample changes, the intensity of this characteristic peak also varies [31]. For instance, as the moisture content of the sample increases, the intensity of the characteristic peak may decrease.

### 3.8. XRD Analysis

XRD is one of the most commonly used methods for studying the crystallization properties of starch and determining the degree of starch crystallization. The effect of OSA modification on the structural changes in TS was analyzed through XRD spectra and changes in crystallization rate. As shown in Figure 4C, the diffraction patterns of TS and O-MTS exhibited the traditional “B-type” with strong peak intensities at 2θ of 5.6°, 15°, 17°, 22°, and 24°. This indicated that the samples had a high content of amylose and similar characteristics of crystal structure. Compared with the TS, with the increase in OSA addition, there was no significant difference in the XRD pattern of the O-MTS, indicating that in the process of OSA modification, the esterification reaction mainly occurred in the amorphous region of the starch, and the crystal structure of the TS had not changed [32]. Zheng et al. [31] also found similar phenomena when modifying ginkgo starch with OSA. Meanwhile, the crystallinity of samples decreased from 32.59% (TS) to 18.39% (O-MTS with 3% OSA addition). The possible reason was that the crystal structure of TS was destroyed in the alkaline system. Zhang et al. [32] also found that OSA modified japonica rice starch resulted in a decrease in crystallinity.

### 3.9. Thermogravimetric Analysis

The -OH groups in the starch molecules can interact, forming hydrogen bonds that allow the formation of crystalline structures between molecular chains. -OH can also form hydrogen bonds with water molecules, thereby affecting the crystal structure of starch. The TGA and DTG curves of TS and O-MTS with different DS values are shown in Figure 4D,E, respectively, where DTG represents the weight loss rate. The results showed that the weight loss process of both TS and O-MTS was divided into two stages.

The weight loss in the first stage was due to the destruction of the binding capacity between starch and water molecules by temperature. In the temperature range of 30–130 °C, the weight loss of TS reached 9.12%, while its mass remained almost unchanged between 130 and 215 °C. At this stage, the weight loss termination temperature of O-MTS was higher than that of TS, about 155 °C. The mass loss increased with the increase in DS value, which were 10.20%, 11.11%, 11.49%, and 13.02%, respectively. This indicated that the water-binding capacity of O-MTS was better than that of TS. The main reason was that the introduction of hydrophilic groups into starch molecules strengthened the interaction between starch and water molecules to a certain extent, resulting in an increase in water-binding capacity.

The second stage was the main period of weight loss. During this period, starch decomposed due to high temperature, resulting in a rapid decrease in its weight. At this stage, the mass of TS lost 57.52%, and the initial temperature of thermal decomposition was about 215 °C. The most rapid mass loss occurred at 285.7 °C. Meanwhile, the initial temperature for the thermal decomposition of O-MTS was about 217 °C, and the fastest mass loss rate occurred at about 301 °C. In addition, the quality loss of O-MTS increased with the increase in DS value (corresponding OSA addition amounts were: 0.5%, 1%, 2%, 3%), which were 62.40%, 63.15%, 63.58%, and 64.86%, respectively, indicating that O-MTS had worse thermal stability. This may be because the introduction of OSA groups further reduced the relative crystallinity of O-MTS; its crystallinity decreased from 32.59% (TS) to 18.39% (O-MTS with 3% OSA addition), and the amorphous area in the particles increased, resulting in the decrease in the thermal stability of starch. Some studies have shown that an increase in the non-crystalline region of starch will lead to a decrease in its thermal stability [32]. This may be because the structure of the crystalline region of starch is stable, while the molecular chains in the non-crystalline region are arranged relatively disorderly, and the intermolecular forces are weaker. When the non-crystalline region increases, it means that the part with relatively strong overall intermolecular forces is reduced, and there is a greater proportion of the part with weaker intermolecular forces in the non-crystalline region. This makes the starch molecules more prone to movement and structural changes, which is manifested as a decrease in thermal stability [31,33]. In addition, from the perspective of moisture, the non-crystalline region exhibits high hydrophilicity and is prone to adsorbing more water, which will also cause the thermal stability of starch to decline at lower temperatures.

### 3.10. The Effects of DS Value on Pickering Emulsion Stability

**Figure 5:**
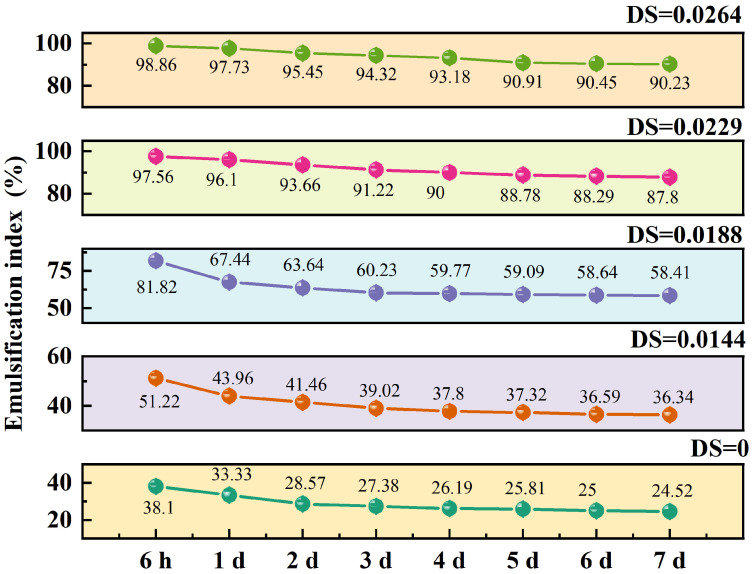
The influence of turmeric starch (TS) and octenyl succinic anhydride-modified turmeric starch (O-MTS) with different degrees of substitution (DS) values on the emulsification index (EI) of Pickering emulsions. Pickering emulsions were prepared under the conditions of a 1% concentration of O-MTS particles and a 1:1 oil–water ratio. The degree of substitution (DS) values of O-MTS were set at 0, 0.0144, 0.0188, 0.0229, and 0.0264, corresponding to OSA additions of 0, 0.5%, 1%, 2%, and 3%, respectively (*n* = 3). This experimental setup aimed to explore how different DS values of O-MTS, along with TS, impact the emulsification index (EI) of Pickering emulsions. “h” represents hour, and “d” represents day.

The EI of Pickering emulsions at different DS values after standing for various times is shown in Figure 5. It could be seen from the table that after a period of placement, the Pickering emulsion with different DS values had decreased to varying degrees. After 7 d of static placement, for the TS (DS = 0), the EI of the Pickering emulsion decreased from 38.10% to 24.52%. Then, the EI of O-MTS (DS = 0.0264) decreased from 98.86% to 90.23%, and its EI increased as the DS increased. This indicates that the Pickering emulsion was better stabilized by O-MTS particles, and the emulsifying property of the Pickering emulsion was improved. Wang et al. [34] also obtained similar results in the study of using OSA-modified corn starch spherulite and potato starch spherulite to stabilize Pickering emulsions.

**Figure 6:**
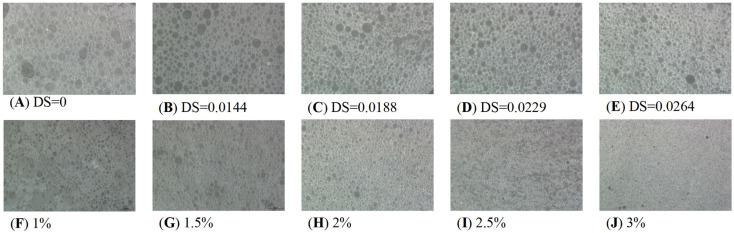
Effects of different degrees of substitution (DS) and starch concentrations on the stability of Pickering emulsions. Pickering emulsions were synthesized under the conditions of a 1:1 oil–water ratio and a 1% concentration of octenyl succinic anhydride-modified turmeric starch (O-MTS) particles. The degrees of substitution (DS) of O-MTS were set at 0, 0.0144, 0.0188, 0.0229, and 0.0264, which, respectively, corresponded to octenyl succinic anhydride (OSA) additions of 0, 0.5%, 1%, 2%, and 3% (depicted as (**A**–**E**) in the figure). The starch concentrations presented in the figure as 1%, 1.5%, 2%, 2.5%, and 3% (shown as (**F**–**J**)) were used to prepare different Pickering emulsions. This experimental design aimed to comprehensively investigate the impact of two key factors—DS values and starch concentrations—on the stability of Pickering emulsions.

The morphology of the Pickering emulsion after 7 d of placement is shown in Figure 6. It can be seen that both the TS and the O-MTS particles can emulsify the oil phase to form a white emulsion phase layer. With the increase in DS value, the height of the emulsified layer of O-MTS stable Pickering emulsion increased. The reason was that esterified O-MTS was hydrophobic and could be adsorbed on the oil-water interface to improve the stability of the lotion [21]. Additionally, the lower layer of the Pickering emulsion was water. It was due to the insufficient amount of starch in the system; only part of the droplets were stabilized, while the rest of the aqueous phase was stratified with Pickering lotion under the action of gravity [35]. Figure 6A–E show the SDM images of Pickering emulsions stabilized by different DS values of O-MTS particles. The Pickering emulsion droplets showed intact and regular spherical shapes. With the increase in DS value, the droplet size of the lotion became smaller, and the droplets became more uniform and dense. However, some large round droplets appeared in the lotion, which may be due to condensation between droplets [30]. Overall, the introduction of the hydrophobic group of OSA made the O-MTS particles have stronger hydrophobicity and surface wettability. In this way, their ability to gather at the water-oil interface will be enhanced, thereby preventing the combination of oil droplets and better stabilizing the Pickering emulsion, greatly improving its emulsifying performance.

### 3.11. Effect of Starch Concentration on the Stability of Pickering Emulsion

**Figure 7:**
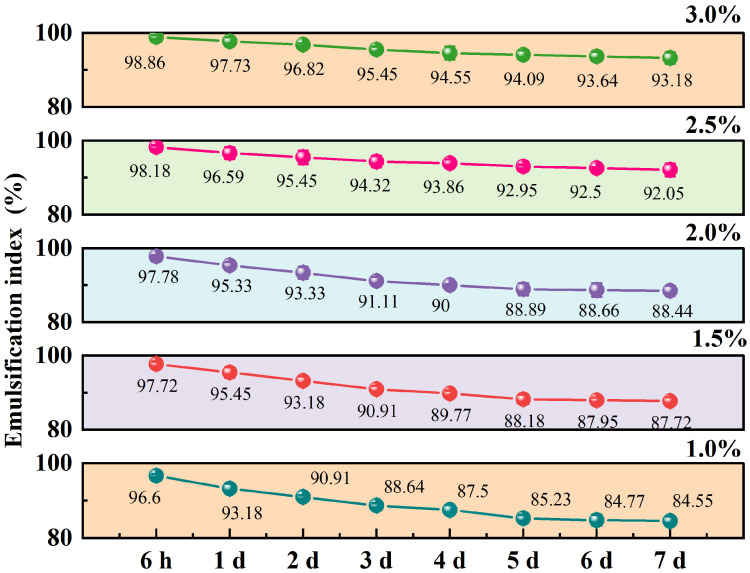
The influence of different starch concentrations of octenyl succinic anhydride-modified turmeric starch (O-MTS) on the emulsification index (EI) of Pickering emulsions. Pickering emulsions were prepared under the conditions of a 1:1 oil–water ratio and a degree of substitution (DS) value of 0.0264 (*n* = 3). The particle concentrations of O-MTS were set at 1%, 1.5%, 2%, 2.5%, and 3% to investigate their effects on the emulsification index of the Pickering emulsions. “h” represents hour, and “d” represents day.

A key indicator of emulsion stability is the particle concentration within the emulsion. Appropriate particle contents play important roles in maintaining the stability of the emulsion and preventing instability, such as creaming and coagulation. The data in Figure 7 indicate that the EI increased with the increase in starch particle concentration. Overall, as the particle concentration increased, the emulsification index exhibited an upward tendency at the identical time point. As time elapses, the emulsification index gradually diminishes under the same particle concentration. Figure 6F–J show the SDM image of the Pickering emulsion with O-MTS particles of different concentrations. With the increase in particle concentration, the droplet size of the Pickering emulsion gradually decreased, the droplet size distribution became more uniform and compact, and the stability of the Pickering emulsion also increased. When the particle concentrations were 1.0% and 1.5%, some large droplets could be observed. When the particle concentration was 2.0%, the emulsion presented round droplets with regular shape but uneven size. When the particle concentration was ≥2.5%, it became finer and more uniform. This was because when the particle concentration was low, a sufficiently tight adsorption layer was not formed at the water–oil interface, resulting in low interfacial tension and causing coagulation and aggregation among droplets, which led to irregularly shaped, unstable droplets [36]. With the increase in particle concentration, the oil–water interface would become increasingly susceptible to the adhesion of starch ester, the EI would increase, the particle size of the lotion would gradually become smaller, and the system would be more stable [37,38].

## 4. Discussion

In this study, O-MTS were prepared and comprehensively investigated. Compared with TS, O-MTS exhibited remarkable improvements in multiple properties. The solubility and swelling degree of O-MTS were enhanced, which could be attributed to the loosening of the molecular structure after OSA esterification modification, facilitating the penetration and interaction of water molecules with starch. This improvement in water-related properties has important implications for its applications in food systems where water-holding and dispersion capabilities are crucial [9,23]. The freeze–thaw stability of O-MTS was significantly better than that of TS. The introduction of carboxyl groups during esterification strengthened the interaction between starch and water molecules, reducing the syneresis rate and the tendency of retrogradation [24]. This property makes O-MTS highly suitable for use in frozen food products, as it can maintain the stability of the food structure during the freezing and thawing processes. Regarding transparency, O-MTS showed higher transparency levels, and this property increased with the increase in OSA. The introduction of carboxyl groups weakened the association between starch molecular chains, reducing light reflection and refraction [25], which is beneficial for applications where the appearance of starch-containing products is important, such as in clear-looking food products or certain cosmetic formulations. In terms of particle-related properties, although the particle shape of O-MTS remained similar to that of TS, its surface became rough (Figure 3), and the particle size increased (Figure 4A). These changes, especially the increase in particle size resulting from molecular aggregation, can affect the physical and chemical properties of O-MTS, including its gelatinization characteristics and light transmittance. Additionally, the water-binding capacity of O-MTS was improved, which is related to the hydrophilic groups introduced by OSA modification, enhancing its ability to retain water.

Regarding the application of O-MTS in Pickering emulsions, significant findings were obtained. The EI of the Pickering emulsion increased with both the increase in DS value and particle concentration of O-MTS. Under the conditions of a DS value of 0.0264 and a particle concentration of 3%, O-MTS could be used to prepare a Pickering emulsion with better performance. From an application perspective, due to the amphiphilic nature of O-MTS resulting from the introduction of carboxyl and alkyl groups, it has great potential as an emulsifier and microcapsule wall material in the food, cosmetics, and pharmaceutical industries. It can be used to improve the stability of emulsions, encapsulate bioactive substances, and control their release, thereby promoting the development of related products and industries. This study thus offers a valuable reference for the in-depth development and utilization of turmeric resources and the modification of other plant starches.

Currently, research on the modification of starch by OSA mainly focuses on corn starch. Cheng et al. [39] modified common corn starch and waxy corn starch with different concentrations of OSA and utilized these modified starches to stabilize emulsions. Compared with common corn starch, OSA-modified waxy corn starch showed a stronger emulsifying capacity. For starches of the same type, the higher the DS, the stronger their ability to stabilize emulsions. Additionally, the OSA-modified starch prepared from high-amylose corn starch as the raw material also exhibits excellent emulsion stability under high-concentration conditions [40]. With the continuous in-depth study of starch sources, some uncommon starches, such as amaranth starch, sago starch, and kudzu starch, have drawn the attention of researchers. Studies have indicated that after the OSA modification of amaranth starch [41,42] and sago starch [28], the starch properties possess good potential in stabilizing emulsions. Zhao et al. [43] compared the abilities of natural kudzu powder and OSA-modified kudzu powder to stabilize emulsions. The results showed that compared with natural kudzu powder, the emulsifying ability of OSA-modified kudzu powder was enhanced. There have been a large number of studies conducted on the stabilization of Pickering emulsions using OSA-modified starches. Besides the DS, which is a crucial determinant, the concentration of OSA-modified starch and the concentration of the oil phase also have a significant impact on the emulsion. Zhang et al. [44] used OSA-modified sorghum starch to stabilize Pickering emulsions and found that the higher the degree of substitution, the better the stability of the emulsion. Dokić et al. [9] studied the concentration of OSA-modified starch in the emulsion and found that as the concentration of OSA-modified starch increases, the droplet diameter of the emulsion decreases, and the stability increases. Overall, our research has similarities with previous studies in some key conclusions. For example, it has been confirmed that the higher the degree of substitution, the stronger the stability of the starch emulsion, and the higher the starch concentration, the stronger its emulsifying ability. Moreover, this research focuses on turmeric starch, a relatively uncommon starch. Our study on the OSA modification of turmeric starch fills the gap in the field of OSA-modified turmeric starch and further enriches the research findings in this area.

## 5. Conclusions

This research centered on octenyl succinic anhydride (OSA)-modified turmeric starches. The optimal process conditions for preparing O-MTS were as follows: reaction time, 5 h; reaction temperature, 40 °C; TS concentration, 35%; pH value, 8; and OSA addition, 3%. Under these conditions, the degree of substitution reached 0.0282. When contrasted with turmeric starch (TS), OSA-modified turmeric starch (O-MTS) demonstrated remarkable improvements in solubility, swelling degree, freeze–thaw stability, transparency, and water-binding capacity. The O-MTS retained a relatively intact morphology, but its particle size slightly increased, and the characteristic peak at 995 cm^−1^ shifted to some extent. The relative crystallinity decreased from 32.59% to 18.39%, and the water binding capacity of O-MTS was improved accordingly. In the context of Pickering emulsions, the emulsification Index (EI) of O-MTS exhibited an upward trend with increasing degrees of substitution (DS) and particle concentration. Specifically, a DS value of 0.0264 and a particle concentration of 3% were identified as the optimal conditions for preparing high-performance Pickering emulsions. These findings significantly deepen our comprehension of turmeric starch modification mechanisms and the stabilization principles of Pickering emulsions. The amphiphilic characteristics of O-MTS endow it with great potential as an emulsifier and microcapsule wall material in the food, cosmetics, and pharmaceutical industries.

## Data Availability

The original contributions presented in the study are included in the article/Supplementary Materials, further inquiries can be directed to the corresponding author.

## Supplementary Materials

The following supporting information can be downloaded at: www.mdpi.com/article/10.3390/foods14071171/s1. Figure S1: The effects of the octenyl succinic anhydride (OSA) addition amount, reaction time, temperature, starch concentration, and pH value on the degree of substitution of turmeric starches and OSA-modified turmeric starches; Table S1: Orthogonal experimental design and results.

## Abbreviations

The following abbreviations are used in this manuscript:

OAS	Octenyl succinic anhydride
TSs	Turmeric starches
O-MTSs	OSA-modified turmeric starches
SEM	Scanning electron microscope
XRD	X-ray diffractometer
FT-IR	Fourier-transform infrared spectroscopy
DS	Degree of substitution
EI	Emulsification index
