# Supplementary material for: The Preparation, Properties, and Characterization of Octenyl Succinic Anhydride-Modified Turmeric Starches and Their Emulsification for Pickering Emulsions
Source: Foods. 2025 Mar 27;14(7):1171. doi: 10.3390/foods14071171 (PMC11989075; doi:10.3390/foods14071171)
Supplement: Supplementary file 1 [file foods-14-01171-s001.zip › foods-3490312-supplementary.pdf]

Supplementary

The Preparation, Properties, and Characterization of Octenyl Succinic Anhydride-Modified Turmeric Starches and Their Emulsification for Pickering Emulsions

Lijuan Fu [1,2,†], Hongfei Chi [1,3,†], Hang Wei [1], Biao Huang [1], Yueyue Qiang [1,3], Mengzhu Shi [1], Ling Fang [1] and Jianwei Fu [1,3,*]

[1] Fujian Key Laboratory of Agro-Products Quality and Safety, Institute of Quality Standards & Testing Technology for Agro-Products, Fujian Academy of Agricultural Sciences, Fuzhou 350003, China; fulijuan_f@163.com (L.F.); chf0825@163.com (H.C.); 13696862957@163.com (H.W.); banbanhb1981@163.com (B.H.); qyydsw@163.com (Y.Q.); mengzhu611@163.com (M.S.); flonly1188@foxmail.com (L.F.)

[2] College of Horticulture, Fujian Agriculture and Forestry University, Fuzhou 350002, China

[3] College of Food Science, Fujian Agriculture and Forestry University, Fuzhou 350002, China

[*] Correspondence: fjw9238@163.com

[†] These authors contributed equally to this work.

S.1 Methods

S.1.1 The extraction of turmeric starch

The turmeric residues remaining after the extraction of curcumin and turmeric essential oil were air - dried under natural conditions, cut into small pieces, and added to distilled water at a solid - to - liquid ratio of 1:4 (g/mL). The mixture was ground in a wall - breaking blender for 60 s. The ground turmeric liquid was then mixed with neutral protease solution (with an addition amount of 0.13% and enzyme activity ≥ 50 U/mg) in a 250 - ml beaker. The pH was adjusted to 6.8, and the mixture was enzymatically hydrolyzed in a water - bath shaker at 52 °C for 6 h. After the enzymatic hydrolysis, it was filtered through a 100 - mesh gauze, and the filtrate was collected. The filter residue was washed with water and filtered three times. The filtrates were combined. The filtrate was centrifuged at a speed of 4500 r/min for 15 min to remove the supernatant. The precipitate was washed with distilled water and centrifuged again at 4500 r/min for 15 min. The dark yellow substances on the surface of the precipitate were scraped off. The precipitate was washed three times with distilled water and centrifuged. Finally, the starch precipitate was dried in an oven at 55 °C with forced air for 12 h to obtain turmeric starch.

S.1.2 Single-factor experiment

(1) The effect of OSA addition on the DS value of O-MTS

Under the conditions of TS concentration was 30%, a reaction temperature of 35℃, a reaction time of 5 h, and a pH of 8.5, the effect of OSA addition (1%, 3%, 5%, 7% ,9%) on the DS value of O-MTS was investigated.

(2) The effect of reaction time on the DS value of O-MTS

Under the conditions of an OSA addition of 3%, TS concentration of 30%, reaction temperature of 35℃, and pH 8.5, the effect of reaction time (2 h, 3 h, 4 h, 5 h, 6 h) on the DS value of O-MTS was investigated.

(3) The effect of temperature on the DS value of O-MTS

Under the conditions of OSA addition of 3%, TS concentration of 30%, reaction time of 5 h, and pH 8.5, the effect of reaction temperature (30℃, 35℃, 40℃, 45℃, 50℃) on the DS value of O-MTS was investigated.

(4) The effect of TS concentration on the DS value of O-MTS

Under the conditions of an OSA addition of 3%, a reaction temperature of 35℃, a reaction time of 5 h, and a pH of 8.5, the effect of starch emulsion concentration (25%, 30%, 35%, 40%,

45%) on the DS value of O-MTS was investigated.

(5) The Effect of pH on the DS Value of O-MTS

Under the conditions of an OSA addition of 3%, TS concentration of 30%, reaction temperature of 35℃, and reaction time of 5 h, the effect of reaction pH (7.5, 8.0, 8.5, 9.0, 9.5) on the DS value of O-MTS was investigated.

S.1.3 Orthogonal experimental optimization

Based on the results of the single-factor experiment, we selected the points with higher DS values in the single-factor experiment results for further experimentation and designed an $L_9(3^4)$ orthogonal test table to study the effects of TS concentration, reaction time, reaction temperature, and pH on the esterification reaction. Thus, the optimal preparation process parameters for O-MTS were obtained.

S.2 Results

S.2.1 The starch content in turmeric

According to the determination by GB/T5009.9 - 2016 "Determination of starch in foods: The second method - Acid hydrolysis method", the average starch content of the turmeric selected in this experiment is 11.05%.

S.2.2 Single factor test results

(1) The effect of OSA dosage on the DS value of O-MTS

From Fig.S1(a), it could be seen that with the increase of OSA addition, the DS value of O-MTS also increased from 0.0184 to 0.0566. At present, the quality requirements for food additives in China and the US FDA had clear regulations. In food grade OSA modified starch products, the amount of OSA added cannot exceed 3.0%. So the dosage of OSA would be controlled at 3.0% in the following single factor experiment.

(2) Effect of reaction time on DS value of O-MTS

Fig.S1(b) shown the relationship between the DS value of O-MTS and reaction time. As the reaction time prolonged, the DS value of O-MTS also increased, reaching a maximum of 0.0290 at 4 h, followed by a gradual decrease in DS value between 4 h-6 h.

(3) Effect of temperature on DS value of O-MTS

According to Fig.S1(c), as the temperature increases, the DS value of O-MTS would increase, with its maximum value occurring at 35℃, which was 0.0282. Subsequently, as the reaction temperature increases, the DS value will show a downward trend.

(4) The effect of starch concentration on the DS value of O-MTS

Fig.S1(d) shown the effect of starch concentration on DS value. From the graph, it could be seen that with the continuous increase of starch emulsion concentration between 25% and 35%, the DS of O-MTS increased from 0.0201 to 0.0285. The DS decreased when the starch concentration was at 40°C and 45°C.

(5) The effect of pH on the DS value of O-MTS

The effect of different pH values on the DS value of O-MTS was shown in Fig.S1(e). When the pH value was between 7.5 and 8.5, the DS value of O-MTS increased with the increase of pH value. When the pH value was 8.5-9.5, the DS decreased with the increase of pH value, and the DS reached a maximum of 0.0276 at pH 8.5.

S.2.3 Analysis of orthogonal experiment results

This experiment used the DS value as the indicator, reaction time, reaction temperature, starch concentration, and pH value as influencing factors, and investigated at three different levels to select the optimal preparation process parameters, as shown in Table S1.

According to Table S1, the order of R values was $R_A > R_B > R_D > R_C$, indicating that the influence of various factors on the DS value of O-MTS was in the following order: reaction time > reaction temperature > pH > starch concentration. The results of the orthogonal experiment showed that the optimal preparation process for O-MTS was $A_3B_3C_2D_2$, with a reaction time of 5 h, a reaction temperature of 40°C, a starch emulsion concentration of 35%, and a pH of 8.5, corresponding DS value was 0.0282. Under these conditions, three sets of validation experiments were conducted, and the average DS value of O-MTS prepared under these reaction conditions was 0.0286, which was higher than the highest value in the orthogonal experiment scheme. Therefore, the optimal process conditions for preparing O-MTS were determined as followed: reaction time of 5 h, reaction temperature of 40°C, starch concentration of 35%, and pH of 8.5.

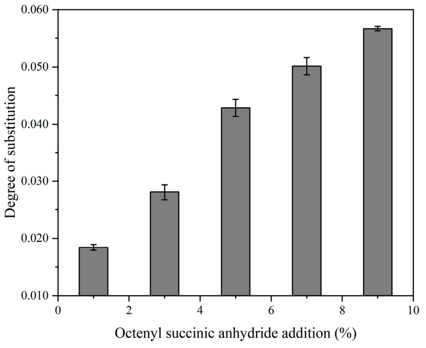

(a)

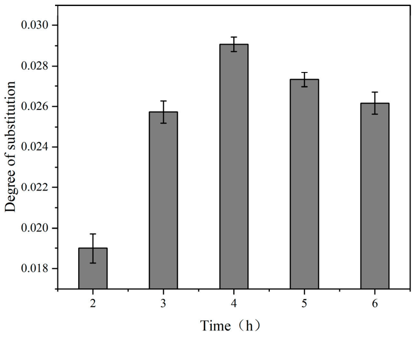

(b)

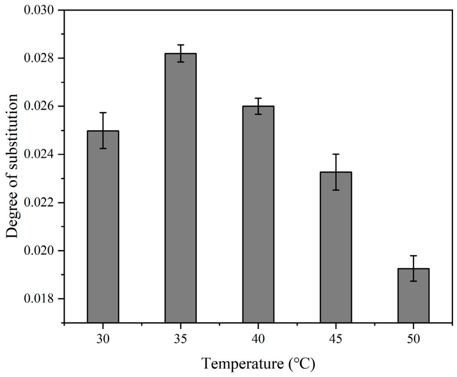

(c)

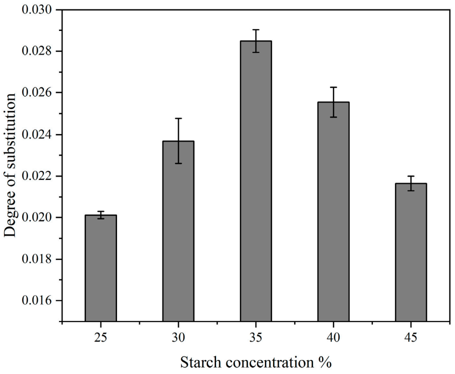

(d)

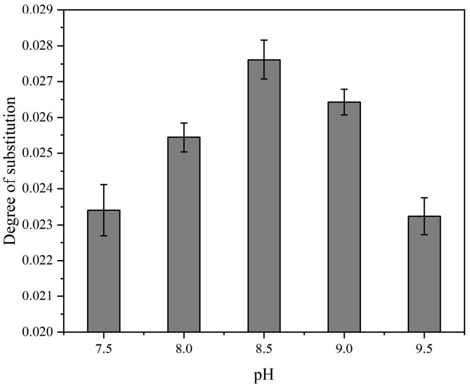

(e)

Figure S1. The effects of the octenyl succinic anhydride (OSA) addition amount (a), reaction time (b), temperature (c), starch concentration (d), and pH value (e) on the degree of substitution of turmeric starches and OSA-modified turmeric starches.

Table S1. Orthogonal experimental design and results

Groups	A:time (h)	B: Temperature (℃)	C: starch concentration (%)	D: pH	DS value
1	3	30	30	8.0	0.0215
2	3	35	35	8.5	0.0216
3	3	40	40	9.0	0.0227
4	4	30	35	9.0	0.0220
5	4	35	40	8.0	0.0219
6	4	40	30	8.5	0.0236
7	5	30	40	8.5	0.0275
8	5	35	30	9.0	0.0272
9	5	40	35	8.0	0.0282
K1	0.0658	0.071	0.0723	0.0716	/
K2	0.0675	0.0707	0.0718	0.0727	/
K3	0.0829	0.0745	0.0721	0.0719	/
k1	0.0219	0.0237	0.0241	0.0239	/
k2	0.0225	0.0236	0.0239	0.0242	/
k3	0.0276	0.0248	0.0240	0.0240	/
R	0.0057	0.0013	0.0002	0.0003	/
